# Does It Measure Up? A Comparison of Pollution Exposure Assessment Techniques Applied across Hospitals in England

**DOI:** 10.3390/ijerph20053852

**Published:** 2023-02-21

**Authors:** Laure de Preux, Dheeya Rizmie, Daniela Fecht, John Gulliver, Weiyi Wang

**Affiliations:** 1 Centre for Health Economics & Policy Innovation, Department of Economics & Public Policy, Imperial College Business School, Imperial College London, London SW7 2AZ, UK; 2 Climate Change & Health Research Unit, Mathematica, Washington, DC 20002, USA; 3 Medical Research Council Centre for Environment and Health, School of Public Health, Imperial College London, London SW7 2AZ, UK; 4 Centre for Environmental Health and Sustainability, School of Geography, Geology and the Environment, University of Leicester, Leicester LE1 7RH, UK

**Keywords:** air pollution, inverse distance weighting, land use regression, pollution exposure

## Abstract

Weighted averages of air pollution measurements from monitoring stations are commonly assigned as air pollution exposures to specific locations. However, monitoring networks are spatially sparse and fail to adequately capture the spatial variability. This may introduce bias and exposure misclassification. Advanced methods of exposure assessment are rarely practicable in estimating daily concentrations over large geographical areas. We propose an accessible method using temporally adjusted land use regression models (daily LUR). We applied this to produce daily concentration estimates for nitrogen dioxide, ozone, and particulate matter in a healthcare setting across England and compared them against geographically extrapolated measurements (inverse distance weighting) from air pollution monitors. The daily LUR estimates outperformed IDW. The precision gains varied across air pollutants, suggesting that, for nitrogen dioxide and particulate matter, the health effects may be underestimated. The results emphasised the importance of spatial heterogeneity in investigating the societal impacts of air pollution, illustrating improvements achievable at a lower computational cost.

## 1. Introduction

There is clear empirical evidence that links short-term exposure to ambient air pollution with a wide range of societal and economic impacts, including on health (e.g., [1,2,3]), productivity (e.g., [4,5,6]), and learning (e.g., [7,8]). However, as pollutants tend to vary spatially and temporally, studies are often challenged by imprecise air pollution estimates to establish such impacts. Air pollutants, such as nitrogen dioxide (${\mathrm{NO}}_{2}$), ozone ($O_{3}$), and particulate matter (PM), originate from different sources, disperse differentially, and can uniquely interact with other environmental factors, such as temperature and humidity, over time. These complexities are further compounded by the computational demands required to model air pollution concentrations at high spatial and temporal resolutions, which makes precise or accurate exposure assessment challenging. Instead, studies often rely on sparse air pollution measurements from monitoring stations and simple assumptions when assigning air pollution exposure to individuals or geographical locations (e.g., schools, factories, hospitals, etc.). As a result, studies may be biased due to measurement error as robust, local, and frequent air pollution levels continue to be difficult to estimate. Thus, the use of air pollution exposures estimated with biases hinders the identification of the air pollution impact on individual outcomes.

In an ideal research setting, individuals would be equipped with personal portable monitors to collect precise and accurate estimates of their exposure as they move across space and time. While this is the most-accurate way of tracking personal exposure, it is extremely costly, cumbersome, mainly available for small samples over a limited amount of time, and not necessarily informative for policy design. In a similar vain, low-cost sensors are suggested as an alternative; but their reliability, availability, and precision are still an issue, and they currently do not support the development of national models. To circumvent these limitations, various exposure assessment methods have been developed to assign air pollution concentrations to a given location (e.g., residential address or hospital). The most-simplistic approach is proximity-based assessments, which are based on the proximity of a location to an emission source or monitoring station to assess changes in ambient air quality [9]. Another approach is spatial interpolation, which generates estimates for unsampled locations using the covariance and distance between the unsampled location and sampled location (e.g., from air monitoring stations). This rests on the principle that near things are more related than distant things [10]. The most-commonly used spatial interpolation techniques is inverse distance weighting (IDW). Although IDW may seem to be an acceptable approach that produces high-frequency time series datasets, it lacks sensitivity to topological variation and atmospheric conditions that may influence some air pollutants. These methods heavily rely on the availability of monitoring data and may produce overly smoothed concentration surfaces, in cases of a limited number of monitoring stations [11]. IDW also has the potential to lead to systematic estimation bias, especially with sparse monitoring networks and topological complexity. On the other hand, dispersion models are mathematical simulations of how air pollutants disperse in the atmosphere. Dispersion models estimate the concentration of pollutants as they travel away from an emission source, how they interact with other pollutants in the atmosphere, and how they are dispersed due to meteorological conditions [12]. Dispersion models are capable of modelling concentrations for short-term (e.g., hourly) and long-term (e.g., annually) averaging periods. The drawback is that dispersion models are demanding both in terms of input data and computational power. In places with no, or limited, air pollution monitoring stations, economists have also explored the use of satellite data (e.g., [13,14]). Satellite information on air pollution can occasionally be obtained at high temporal frequency; however, its use requires expensive pre-processing, and data are not often available at the required spatial resolution [15]. Its accuracy is dependent on the spatiotemporal characteristics of the air pollutant considered. The availability of satellite sensors is disproportionately spread globally, increasing the difficulty in studying low- and middle-income settings. Therefore, such estimates are often not readily available nor easily accessible to social science researchers.

Economists interested in the impact of air quality on societal outcomes often develop economic models using natural experiments or simple exposure assessment methods. Natural experiments rely on an exogenous change in emission sources (e.g., the closure of factories or a change in government policies) to overcome measurement challenges and avoid the need to accurately quantify changes in air pollution concentrations. While this method might be able to uncover causal relationships, it is not suitable to establish concentration–response relationships. Therefore, to assess concentration–response relationships, economists tend to employ simple exposure assessment methods (e.g., nearest-neighbour matching or IDW). These methods sacrifice either the temporal frequency by relying on annual averages or the geographical precision by assigning the same air pollution level to a large number of locations.

In this paper, we propose a simplified exposure assessment approach to produce temporally and spatially highly resolved estimates for the main regulated air pollutants: nitrogen dioxide (${\mathrm{NO}}_{2}$), ozone ($O_{3}$), and particulate matter with a diameter smaller than 10 µm (${\mathrm{PM}}_{10}$) and smaller than 2.5 µm (${\mathrm{PM}}_{2.5}$). Our method relies on land use regression (LUR) models to derive robust estimates of local air pollution levels. Compared to dispersion models, LUR models are less challenging in terms of input data and computational processing and can account for high spatial variability. With their relatively low demand on the input data, LUR models have the potential to provide an improved, yet accessible, robust alternative to weighted averages whilst capturing the spatial heterogeneity of air pollution. Traditional LUR models are widely used in predicting long-term (e.g., annual) air pollution estimates. However, since typical land use input variables (e.g., road distribution, population density, etc.) are fairly constant over time, their application to estimate short-term (e.g., daily) exposures is limited. (Therefore, they are commonly used to develop annual models as the variables (e.g., land use, road length) are time-invariant. It is, in principle, possible to develop daily LUR models, but the lack of daily data required to build the model is generally a restriction. Over the last decade, there has been increasing interest in combining different modelling techniques to overcome their respective limitations, so-called “hybrid models”. Our methodology accounts for both environmental characteristics that may influence emission and dispersion patterns and daily variability. This approach relies on LUR and allows for the derivation of estimates at a fine geographical scale, as well as at a high time frequency, which increases the accuracy compared to the standard IDW.

To illustrate the effectiveness of this approach, we developed daily air pollution estimates (*daily LUR*) across England. We validated the models in space and time using an independent subset of data from the monitoring stations. Similarly, we estimated the weighted averages of pollution measurements using IDW. We assigned both our daily LUR and IDW estimates to hospitals in the National Health Service (NHS) in England and assessed the impact of the daily variation of air pollution on accident and emergency (A&E) visits between 2010 and 2011 using a flexible multiple fixed effects distributed lag model [6,16]. This allowed us to quantify the impact of different air pollution exposure assessment models on health outcomes. The differences incurred by exposure assessments may subsequently influence policy perspectives.

Our results varied by pollutant. ${\mathrm{NO}}_{2}$, ${\mathrm{PM}}_{2.5}$, and ${\mathrm{PM}}_{10}$ demonstrated notable discrepancies between the two exposure assessment approaches—with daily LUR estimates resulting in statistically significant effects, while IDW estimates suggesting no impact of air pollution on A&E visits. The effect sizes using IDW were half those estimated by daily LUR. Conversely, health estimates from $O_{3}$ were similar when using IDW measurements and daily LUR estimates.

Our findings suggest that the use of IDW risks the introduction of a substantial downward bias, which has the potential to limit the ability of uncovering potential economic estimates and underestimate the potential effects of air pollution. This paper proposes a simpler methodology to improve the accuracy of assigning air pollution exposure across space for studies that require temporally high-resolution information (e.g., daily or weekly). It should be clear that the daily LUR is not the panacea to pollution exposure and that there are more complex methods to assign pollution exposure, for example using machine learning (e.g., random forest, XGBoost, neural networks). However, they require more data and are computationally intensive. Therefore, the daily LUR represents a user-friendly improvement over the IDW method.

The remainder of the paper is structured as follows: The next section presents a brief background of air pollution assessment techniques (Section 2). Section 3 presents our proposed exposure assessment technique. Section 4 applies this technique to a case study, outlining our health setting and empirical approach. Findings from this case study are reported in Section 5 and compare estimates between the techniques. Finally, Section 6 discusses the implications of our findings and concludes.

## 2. Background on Air Pollution Exposure Assignment

Air pollution is one of the most-serious environmental concerns of our generation: not only is it closely linked with anthropogenic activities related to climate change, it also directly affects individuals’ health and well-being. Air pollution has given rise to extensive research documenting increased mortality (e.g., [1,2,3]) and morbidity (e.g., [17,18]) and decreased productivity and human capital (e.g., [4,5,6]) and school performance (e.g., [7,8]). Given the complexity in accurately estimating air pollution levels, economic studies often have to make trade-offs between temporal and spatial precision in estimating air pollution or circumvent estimating air quality altogether. Approaches can be broadly categorised into four types of air pollution studies on economic outcomes: studies using (i) specific sources of air pollution (e.g., emissions from a factory), (ii) natural experiments that provide a rapid exogenous change in the ambient air quality (e.g., policy changes), (iii) air pollution modelling (e.g., modelling air quality with satellite-based products in [19]), and (iv) monitoring stations capturing specific pollutants at a specific location.

The first approach utilises variation in specific sources of air pollution, such as emissions from traffic (e.g., [20,21,22]) or manufacturing sites (e.g., [23]). In these studies, the impact of ambient air quality is only indirectly captured by a relative change of activity at the source. The main issues with this approach are that it only captures the effect of a unique variation in a local source, often without knowledge of its impact on the overall air quality, and it assumes that the emissions from other sources (e.g., manufacturing sector) remain constant over the period of the evaluation. Additional assumptions on the spatial extent of impacts are also required. While it may serve to demonstrate that a change in air pollution is beneficial or detrimental to the outcome of interest, this approach cannot inform dose–response relationships and peaks of air pollution (e.g., [21,24,25]).

Secondly, natural experiments or quasi-experimental approaches (e.g., [3,24,26]) are commonly adopted and focus on abrupt, and often unanticipated, changes in ambient air pollution levels. These research designs typically come from changes in environmental policy, such as the introduction of the 1970 Clean Air Act (e.g., [3,24,27]) or the closure of power plants (e.g., [28]). The advantage of this approach is that it controls for the issues of residential sorting, as well as acclimatisation. The former refers to the possible bias from individuals choosing their residential location as a function of ambient air quality and their individual susceptibility to, or preference for, air quality. A natural experiment typically offers an abrupt change in air quality, and the observed effect is more likely to be a result that can be attributed to the change in air quality as opposed to behavioural changes, such as avoidance behaviour (see [17,29,30] for a discussion on *avoidance behaviour*). Due to this, it could be argued any detected effects are close to causal. However, it only relies on a single and local variation in average air quality across a specific population and often does not capture daily changes in air pollution concentrations.

The third approach, using annual air pollution models, predicts spatially granular estimates, via data-intensive and complex models, but is only feasible for annual estimates due to the complexity of the models [31]. This presents an attractive option to researchers due to its ability to provide air pollution at a fine granular scale that captures the heterogeneity of a pollutant’s geographical distribution (e.g., [32]). However, as estimates are often limited to long-term annual averages, they fail to account for the burdens imposed by short-lived pollutants (e.g., ozone) and prevent one from obtaining short-term variations that may have separate effects on the outcomes of interest (e.g., health and academic performance [33]).

Finally, economists also use direct measurements from ground monitoring stations (e.g., [6,16,34]) or satellites (e.g., [35,36]). Monitoring stations are becoming increasingly prevalent, particularly in urban locations. They represent a valuable source of information, often on an hourly basis, that captures temporal changes at their location (e.g., [37] with ${\mathrm{SO}}_{2}$ and black smoke, [1,38,39,40] with CO, ${\mathrm{PM}}_{10}$, and $O_{3}$,^1^] with ${\mathrm{PM}}_{10}$ and $O_{3}$, and [2] with ${\mathrm{PM}}_{2.5}$ and $O_{3}$). Such time series data are easy to obtain and straightforward to analyse; however, air pollution monitoring networks often remain sparse, and assumptions are required to obtain proxies of local air pollution concentrations in areas without monitoring stations. A common approach has been to assign a point of interest, an area, or an individual to their nearest monitoring station (nearest neighbour matching). However, this has been shown to be a poor marker in spatial assessments of air pollution exposures [41] as it disregards the various essential dispersion characteristics of each pollutant. Another naive practice has been to average the stations’ values across the neighbourhood of the points of interest [42,43,44]. While this offers frequent estimates, its geographical aggregation, similar to nearest neighbour matching, is likely to introduce a large bias as it does not account for the regional characteristics that affect air pollution sources and dispersion.

There is growing interest in exploring the effects of, and accounting for, short- and long-term variation in air pollution without sacrificing spatial granularity. On the one hand, advanced modelling, in principle, could achieve higher temporal frequency, but requires high computational power and more input data than are often available. These models are often challenging to implement across a large geographical area (e.g., across a country) or over a long time period. On the other hand, daily or hourly air pollution measurements are easily accessible given the wide availability of monitoring stations, but suffer from systematic biases when the pollutant’s dispersion characteristics and local topography are not accounted for. Therefore, these limitations present a need for an approach in air pollution exposure assignment that is both (1) accessible to social scientists, amongst other disciplines, and (2) considerate to the range of influencing factors of each pollutant, which enables a more accurate air pollution assignment to provide robust evidence of the impacts of air pollution concentrations on a wide range of outcomes.

## 3. Methodology

Our modelling approach was based on an LUR model, which is a widely used air pollution exposure assessment method to estimate annual average air pollution concentrations for environmental epidemiology [45,46,47]. LUR models have been developed for cities in North America [48], Europe [49], Asia [50], Australia [51], South Africa [52], and larger geographic areas including North America [53,54], Europe [55,56], Australia [57], and Asia [58]. The spatial resolution of LUR models provides the opportunity for estimates on a fine geographical scale, depending on their land use variables—typically ranging from 100 m by 100 m to 1 km by 1 km. In order to obtain air pollution estimates of more frequent temporal variation (i.e., daily), we propose temporal scaling of the traditional LUR (*daily LUR*). This approach offers a more accessible and reliable way of estimating daily ambient air pollution in various geographical settings as opposed to predictions derived purely from empirical relationships.

We modelled the annual LUR model using a standard methodology and detail its steps in Appendix A. This begins by gathering air pollution measurements at monitoring stations, then identifying variables that can (a) predict the measured air pollution concentrations from various sources (e.g., road traffic and industrial plants) and sinks (e.g., forests) and (b) estimate the direction of their effects using a regression model. We combined the traditional land use input variables with a chemical transport model (CTM). The CTM estimates simulate the physical and chemical processes of pollutant transport based on emission inventories (location, strength, size) and meteorological inputs (e.g., temperature, relative humidity, wind speed, and wind direction). The model was then calibrated and validated against data from monitoring stations, before the production of concentration surfaces.

The daily LUR estimates proposed in this paper were derived as follows: once validated annual surfaces were obtained, the annual estimates were scaled to obtain temporal variation using measurements from air pollution monitoring stations. The granularity depends on the requirements of the study. For example, one may have individual health data geocoded at the level of a geographic unit *p*. Supposing we have LUR estimates for *N* geographic units, *p*, we take the centroid of each unit and assume the centroid $p_{c}$ to be representative of the entire unit. Modelled annual air pollution concentrations $C_{annual}^{\prime }$ are extracted from the LUR surface at all geographic unit centroids. Daily exposure for each *p*, $DailyExpo_{p_{c}}$, is calculated by scaling monitored daily concentrations to annual concentrations such as

(1) $$DailyExpo_{p_{c}}=\frac{C_{daily}}{C_{annual}}\times C_{annual}^{\prime }$$

where $C_{daily}$ and $C_{annual}$ are the measured daily and annual concentration from the nearest background monitoring station of the geographic unit centroid $p_{c}$, respectively; $C_{annual}^{\prime }$ is the estimated annual concentration of each geographic unit extracted from the LUR surface.

In practice, it is unlikely that the outcome data and LUR estimates are at the same geographical scale. If outcome data are geocoded for an aggregated area, *q*, which is larger than *p*, the annual concentration for each *q*, $AnnualExpo_{q}$, is calculated by averaging all modelled annual air pollution concentrations $C_{annual}^{\prime }$ for each *p* within each *q* (Equation (2)), again assuming the centroid $q_{c}$ to be representative of the entire area *q*. Daily exposure for each $q_{c}$, $DailyExpo_{q_{c}}$, is then calculated using the aggregated annual exposure (Equation (3)).

(2) $$AnnualExpo_{q}=\frac{\sum C_{annual}^{\prime }}{N}$$

(3) $$DailyExpo_{q_{c}}=\frac{C_{daily}}{C_{annual}}\times AnnualExpo_{q}$$

where *q* is the geographic area, $C_{annual}^{\prime }$ is the estimated annual concentration of each geographic unit extracted from the LUR surface, *N* is the number of geographic units, *p*, within each aggregated area *q*, and $C_{daily}$ and $C_{annual}$ are the measured daily and annual concentration from the nearest background monitoring station of geographic unit centroid $q_{c}$, respectively.

## 4. Case Study: A&E Visits to the English National Health Service

To assess the performance of the daily LUR model, compared to IDW, we applied the air pollution exposure assignment approach described in Section 3 and IDW to a healthcare setting in England. We modelled A&E visits to hospitals in the NHS across England from 1 April 2010 to 31 March 2011 as a function of air pollution assigned to the neighbourhood of the hospital, controlling for various confounders. A&E visits do not require a diagnosis, and therefore, the majority of the visits are unclassified in terms of disease or visit purpose. We began by quantifying the differences between air pollution concentrations from different exposure assignment techniques. We subsequently quantified the differences in the estimated air-pollution-associated A&E visits using the two different exposure assignment techniques. This allowed us to illustrate how the use of daily LUR estimates performs against IDW estimates when identifying its impact on social outcomes.

### 4.1. Study Population and Data Sources

All observations were unique at the day and hospital level with the sum of A&E visits to the hospital on that day. All observations were then assigned an air pollution concentration using daily LUR and IDW to the centroid of the hospital postcode district (PCD) level. Further, they were also assigned meteorological characteristics measured from the nearest monitoring station, including important confounders, temperature, and relative humidity. We matched all data at the hospital visit date level between 1 April and 31 March 2011. Summary statistics describing our air pollution data can be found in Appendix B. Below, the emphasis is on further detailing each dataset implored in our empirical illustration.

The Automatic Urban and Rural Monitoring Network (AURN) [59] provides ratified daily mean measured concentrations of four major health-relevant pollutants: ${\mathrm{NO}}_{2}$, $O_{3}$, ${\mathrm{PM}}_{2.5}$, and ${\mathrm{PM}}_{10}$. The AURN classifies monitoring stations as background urban, background suburban, background rural, traffic urban, industrial urban, and industrial suburban. We only included background sites to avoid the influence of road traffic and industrial emissions, which can result in biased exposure assignment (i.e., overprediction). The completeness of the data was checked for each pollutant and each monitoring station based on a 75% completeness site selection rule. A monitoring station was included if it had more than 75% daily mean measurements over (a) an entire year and (b) within each month. This site selection rule ensures that the available daily data of each site have a good representativeness of a year when they are averaged for the annual mean. For ${\mathrm{PM}}_{2.5}$ and ${\mathrm{PM}}_{10}$, this selection rule resulted in too few sites; therefore, a less-stringent criterion of 50% data completeness applied for these two pollutants. After applying the above criterion, the number of selected monitoring stations used in Equation (A1) (Appendix A) over this study year was 59 for ${\mathrm{NO}}_{2}$, 63 for $O_{3}$, 38 for ${\mathrm{PM}}_{2.5}$, and 25 for ${\mathrm{PM}}_{10}$.

The meteorological data, which are used as confounders in Section 4.5, came from the Met Office Integrated Data Archive System (MIDAS) database. It provides meteorological characteristics collected by the Met Office. The meteorological conditions aspects are captured by irregularly spaced stations across England. The dataset contains daily and hourly meteorological measurements, such as daily air temperature and relative humidity, provided by 106 stations.

A&E visits came from the Hospital Episode Statistics (HES) database from NHS Digital across 220 hospitals in England. These are the universe of visits over that period. The mean number of daily A&E visits was 200 (SD 124) per hospital. The mean age of all visits was 37.9 (SD 6.6) years old, with 48.8% of patients being female. The data provide information on hospital utilisation. Each observation includes details on visit type (e.g., treatment, diagnosis type), socioeconomic status (Index of Multiple Deprivation), patient characteristics (e.g., age group and gender), and hospital specifics (e.g., postcode). Data are collected during a patient’s visit to the provider for multiple administrative and financial purposes. Due to a high rate of missing values in the classification of diagnoses or treatments, we used all-cause A&E visits.

### 4.2. Pollution Assignment Methods

We began by estimating an LUR model for England and then applying the methodology outlined in Section 3. For our LUR model, we obtained six types of Geographic Information System (GIS)-derived land use data including: land cover, population/household, road network, traffic, topography, and building. The predictors were chosen mostly based on the ones used in the European Study of Cohorts for Air Pollution Effects (ESCAPE) study [60], with one predictor on building volume as a proxy for street ventilation [61]. When estimating our annual air pollution surface (Stage 3 in Appendix A) to derive daily LUR estimates, we used a resolution of 25 m by 25 m because it is the smallest resolution of the datasets (i.e., land cover). At this resolution, the spatial variation of the variables is not aggregated. However, as previously mentioned, the resolution can be adjusted according to the study needs. Given the size of the selected monitoring stations, we used a 5-fold cross-validation. This allowed an adequate amount of sample data to be included in each fold and used in the validation. Models are summarised by several measurements including the adjusted $R^{2}$, root-mean-squared error (RMSE), and coefficient (β) in Appendix B Table A1.

We then implemented the strategy outlined in Section 3 to obtain air pollution estimates by daily LUR. We applied the scaling in Equation (3) to an LUR model (described in Appendix A) for 2778 PCDs across England over the same period. For our application, we used the centroid of each PCD. Air pollution estimates were assigned to each NHS hospital using their PCD. Air pollution was assigned at the hospital level as this analysis looked at the contemporaneous impact on A&E visits. A&E visits capture the immediate effects of deviations in air pollution levels. As the average distance of a patient’s residential postcode district to his/her A&E hospital is 13.3 km (SD 10.9), with a maximum distance of 223 km, the assignment of air pollution at the hospital level reduces the risk of inaccurately assigning location exposure.

As a comparison, a spatial interpolation of monitoring data using IDW is included. IDW does not involve statistical modelling: it is based on the distance weighting of nearest monitoring stations to a location. Air pollution exposure at each PCD *j* on day *t*, $DailyExp_{tj}$, equals the average values of daily measurements from *k* monitoring stations within a 50 km radius, with weights proportional to the inverse of the square of the distance between their residence and the monitoring station (Equation (4)).

(4) $$DailyExp_{tj}=\sum_{k=1}^{n}P_{kt}\times \frac{\frac{1}{d_{kj}^{2}}}{\sum_{k=1}^{n}\frac{1}{d_{kj}^{2}}}$$

where $P_{kt}$ is the measured daily concentration at each monitoring station *k* on day *t*; *d* is the distance between postcode centroid *j* and monitoring station *k*. We used a maximum radius of 50 km to include a moderate number (*n*) of monitoring stations. For instances where a PCD has no monitoring stations within 50 km, we used the nearest monitoring station (n=1).

### 4.3. Defining Air Pollution Bins

The primary exposure variables of interest in this analysis were seven 5 $\mu gm^{-3}$ daily air pollution bins, constructed for ${\mathrm{PM}}_{2.5}$ and ${\mathrm{PM}}_{10}$, ranging from values under 5 $\mu gm^{-3}$ to over 30 $\mu gm^{-3}$. For ${\mathrm{NO}}_{2}$, six 10 $\mu gm^{-3}$ daily air pollution bins were constructed ranging from values under 10 $\mu gm^{-3}$ to over 50 $\mu gm^{-3}$. Finally, seven 10 $\mu gm^{-3}$ daily air pollution bins were constructed for $O_{3}$ ranging from values under 10 $\mu gm^{-3}$ to over 60 $\mu gm^{-3}$. These thresholds were used to ease the comparison of the air pollution assessment methods. These variables indicate whether air pollution measured at a given NHS hospital falls in the specified air pollution range. As daily air pollution is defined at the NHS hospital level (hospital-day), we preserved the spatial variation in air pollution to allow for the identification of its effects. The 0–10 $\mu gm^{-3}$ bin (${\mathrm{NO}}_{2}$ and $O_{3}$) and 0–5 $\mu gm^{-3}$ bin (${\mathrm{PM}}_{2.5}$ and ${\mathrm{PM}}_{10}$) were the reference categories and omitted in all regressions; consequently, all estimates were interpreted as the impact of a day in the given air pollution range relative to a day in either the 0–5 $\mu gm^{-3}$ or 0–10 $\mu gm^{-3}$ range.

### 4.4. Quantifying the Differences in Exposure Assessment Approaches

In this section, we compare air pollution estimates derived from different air pollution exposure assignment methods (daily LUR and IDW) and any differences that may be subsequently introduced in air pollution–health impact analysis. We first compared annual air pollution concentrations spatially through maps to illustrate the geographical variation in air pollution concentrations. Second, we compared the correlations of daily estimated air pollution concentrations with observed values (i.e., air pollution measurements) at monitoring stations that we used as a benchmark to quantify the potential bias introduced by daily LUR and IDW. Our third comparison was similar to the second, but using annual levels, we randomly omitted some air pollution monitoring stations to derive the air pollution estimates at the monitoring station and compared the derived estimates to actual measurements taken at these locations. Finally, we describe how air pollution exposure assigned to hospitals was classified into different treatment bins—potentially creating different treatment intensities and, thus, impacting the overall conclusion.

**Figure 1:**
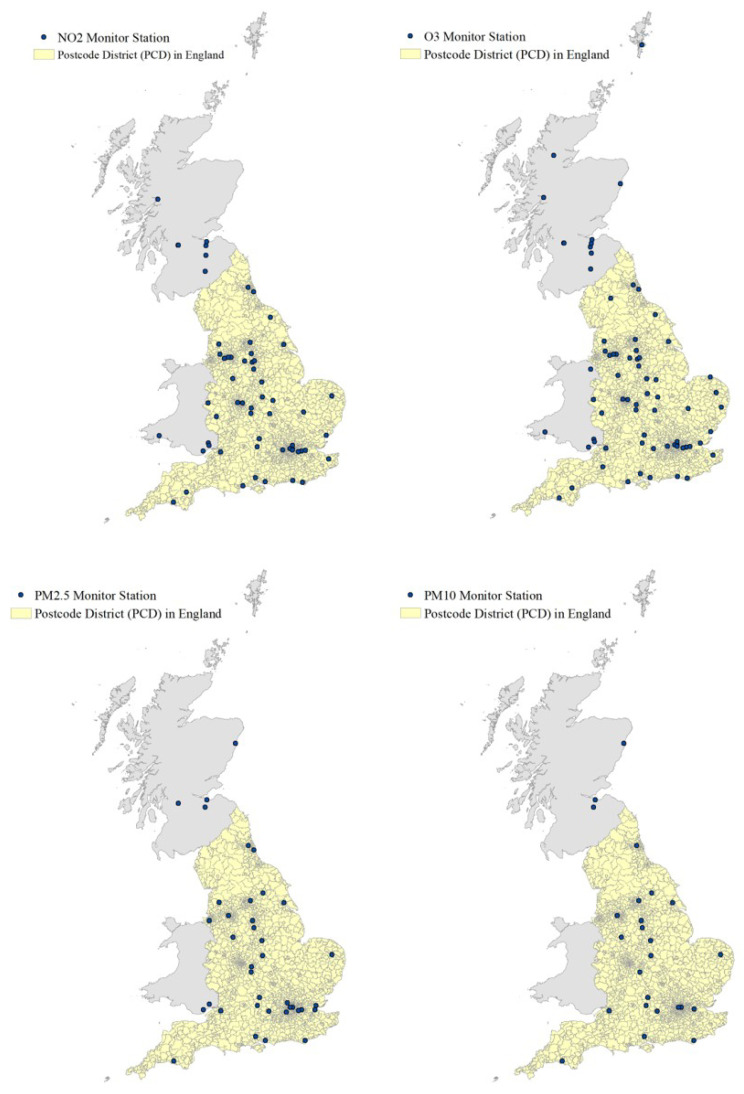
Spatial distribution of monitoring stations across Great Britain

The spatial distribution of the monitoring stations for ${\mathrm{NO}}_{2}$, $O_{3}$, ${\mathrm{PM}}_{2.5}$, and ${\mathrm{PM}}_{10}$ is shown in Figure 1. We included stations from Wales and Scotland to “borrow” measurements from monitoring stations, within 50 km of England. We produced air pollution surfaces at the PCD level to visually compare the spatial pattern generated from the two approaches (daily LUR and IDW). The comparison of the maps provides an insight into the spatial heterogeneity of the different methods. Greater spatial granularity enables the identification of hot spots of air pollution, which are often in densely populated areas (e.g., London, Birmingham) and, therefore, essential to assess the impact of air pollution on individuals.

Whilst the comparisons of daily air pollution estimates are insightful, they are not representative of the precision of IDW in other locations, as the comparisons occurred at monitoring stations where the IDW estimates were calculated from. In order to assess the precision of IDW, we further explored the magnitude of any discrepancies through a “bench-marking” approach. The measurements at monitoring stations (*MON*s) were considered *true* observations of air pollution exposure that we can use to compare against air pollution estimates derived from the other exposure assignment methods. Specifically, we were interested in the difference between these true observations and the concentrations derived using assignment methods. We applied the daily LUR model and IDW to obtain daily air pollution estimates, for all four pollutants, at each monitoring station, while excluding the station in question from its own measurement/estimation. For example, for IDW estimate at monitoring station *A*, we deliberately excluded measurements from *A* and used measurements from the second-nearest station, *B*. This was to mimic situations where a location for estimation is not near a monitoring station. We then calculated the absolute difference ($Deviation_{mt}$) at the monitoring station, *m*, following

(5) $$|Deviation_{m,d}|=|Pollution_{m,d}^{i}-Pollution_{m,d}^{MON}|$$

where $Pollutant_{m,d}^{i}$ is the air pollution concentration at the monitoring station, *m*, on day *d*, estimated through air pollution technique *i*. *i* can be from: IDW or daily LUR. $Pollutant_{m,d}^{MON}$ is the average daily air pollution concentration reported at monitoring station *m* on day *d*. The same bench-marking calculations were conducted using IDW, daily LUR, and satellite monitors (SAT) at an annual level for ${\mathrm{NO}}_{2}$ and ${\mathrm{PM}}_{2.5}$. Annual calculations were estimated as this was the most granular temporal scale available using satellite monitors. These results mirror the results that are presented in the paper with a larger deviation observed for SAT for ${\mathrm{NO}}_{2}$.

To compare daily estimates from the daily LUR and IDW, as described in Section 4.2, we produced pairwise scatter plots that compare daily estimates against measurements recorded from monitoring stations. We used the Pearson correlation coefficient (Pearson’ r) to indicate the strength of the linear relationship between the two sets of data.

In addition, the accuracy and precision of the models were quantified by regressing daily predictions (from the “bench-marking” approach, where values from the station in question were excluded) against daily measurements and summarised in terms of the coefficient of determination (*R*${}^{2}$), root-mean-squared error (RMSE), beta, and intercept.

It could be argued that the conventional modelling approach in social sciences, by flexibly modelling air pollution impacts through the use of indicator bins, small deviations in concentrations from different air pollution exposure methods are unimportant should observations be properly classified into correct indicator bins. However, mismeasurement of an individual’s or unit’s air pollution exposure risks misclassification of individuals to indicator bins. To assess how the classification varies across the different methods, we compared any deviations between the assigned bins from daily LUR and the IDW for each observation by calculating the percentage of observations that did not fall into the same indicator bin category.

### 4.5. Identification Strategy

To identify the effects of each pollutant, we exploited the panel structure of our data and built on the panel approach used in [6,16,62]. We introduced NHS hospital fixed effects (FE), which account for local air quality baselines and allowed us to identify the impact of short-term air pollution variation around the local average air quality. Implicitly, NHS hospitals without high peaks of air pollution throughout the year form a counterfactual for NHS hospitals that do have peaks in that same year, after accounting for fixed differences between the NHS hospitals and for common time effects. Naturally, many hospitals had multiple events over the period of the analyses. An attractive feature of this approach is that it builds in placebo tests that should identify likely violations of this assumption. Furthermore, this identification strategy relies on the unpredictable and presumably random daily local variation in air pollution.

Using a panel dataset, we employed a distributed lag Poisson regression model with multiple fixed effects to estimate the effect of air pollution on daily A&E visits and for the three days following a day in which air pollution falls into an extreme air pollution bin. Equation (6) denotes the reduced form relationship between air pollution and A&E visits. The total net effect of air pollution on A&E visits was flexibly modelled by including a series of indicator variables for air pollution.

The goal was to estimate the net effect of air pollution on day *d* on the number of A&E visits ($Y_{jd}$) per NHS hospital, *j*, per day, *d*, and for three days following day *d*:

(6) $$\begin{array}{c} log\left(Y_{jd}\right)=\alpha +\sum_{p\in (<b,\ldots >u)}\beta_{p}Pollutant_{jd}^{p}+\beta MeanTemp_{jd}+\iota Humidity_{jd}+\\ +\sum_{l=1}^{3}\pi_{p_{1}l}Pollutant_{jl}^{p_{1}}+\sum_{l=1}^{3}\pi_{p_{2}l}Pollutant_{jl}^{p_{2}}+\sum_{l=1}^{3}\pi_{p_{3}l}Pollutant_{jl}^{p_{3}}+\\ \zeta_{k}DayofWeek_{k}+\rho_{r}Holidays_{r}+\sigma_{m}Month_{m}+\tau_{j}Hospital_{j}+\epsilon_{jd} \end{array}$$

$Pollutant_{jd}^{p}$ are a series of regressors that equal 1 if the daily air pollution at NHS hospital *j* falls into a predefined air pollution bin and zero otherwise. For each pollutant, regressions were run separately using the air pollution bins described above. Consequently, these coefficients $\beta_{h}$ semi-parametrically describe the pollution–visits relationship, the net of seasonal influences and relative to the lowest air pollution bin (i.e., 0 $\mu gm^{-3}$ to 5 $\mu gm^{-3}$ or 0 $\mu gm^{-3}$ to 10 $\mu gm^{-3}$) that is omitted in all regressions.

$Pollutant_{jl}^{p_{1}}$, $Pollutant_{jl}^{p_{2}}$, and $Pollutant_{jl}^{p_{3}}$ are indicator variables for up to 3 days following a day in a predefined air pollution bin of extreme air pollution exposure and zero otherwise. Therefore, the extreme air pollution lag effect was estimated for 30 days following a day of extreme air pollution.

A&E visits, health, and air pollution vary seasonally. A series of time-fixed effects for day of the week ($DayofWeek_{k}$), school and bank holidays ($Holidays_{r}$), and month ($Month_{m}$) intended to control for the seasonal effects of cyclical variation. The use of time-fixed effects makes no assumptions on seasonal form, does not constrain the model, and avoids specification errors. Additionally, as seasonality is measured at a relatively fine scale, the flexibility inherited from such granular fixed effects also accounts for health changes that are driven by long-term behavioural changes. In addition, fixed effects for NHS hospitals were also included for 220 NHS hospitals over our study period ($Hospital_{j}$). As our observed geographical unit was the NHS hospital, the inclusion of these fixed effects also captures population grouping effects, such as residential sorting. Overall, these variables account for the influence of unobserved confounding factors.

$MeanTemp_{jd}$ and $Humidity_{jd}$ represent the daily mean temperature (in Celsius) and daily relative humidity on day *d* at NHS hospital *j* and were included as potential confounders of the effect of air pollution on A&E visits. Finally, $\epsilon_{jd}$ represents the standard idiosyncratic disturbance term.

We used clustered and robust standard errors to allow for arbitrary within-group correlations at the hospital level. All analyses were conducted with Stata MP v15 [63].

## 5. Results

### 5.1. Quantifying the Differences in Exposure Assessment Approaches

**Figure A1:**
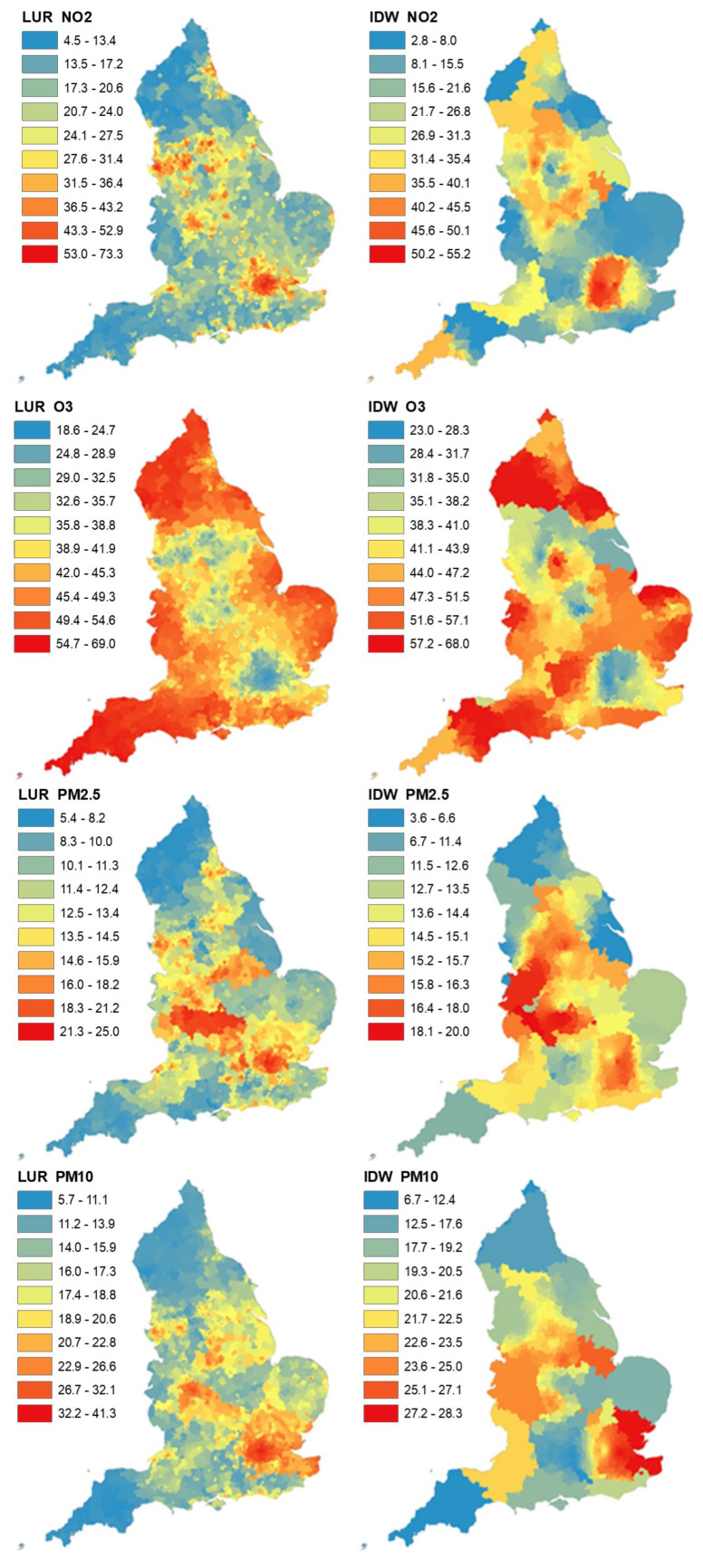
Annual average air pollution concentrations of the LUR model across England, over the year of interest, at the postcode district level (**left**); annual average of IDW of one to four nearest monitoring stations at the postcode district level (**right**). Concentration displayed in $\mu gm^{-3}$.

**Figure A2:**
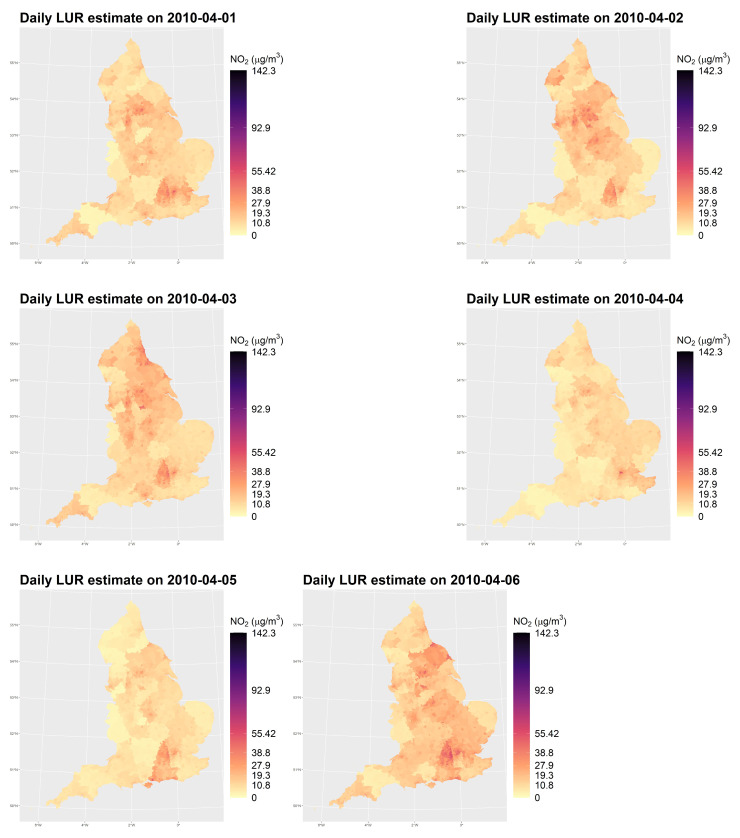
Daily ${\mathrm{NO}}_{2}$ surfaces from 1 April 2010 to 10 April 2010, produced from the daily LUR model. The spatial unit is the postcode district. The maps demonstrate spatial variation across England and temporal variation across six consecutive days. For example, 6 April 2010 shows an elevated pollution level in most regions.

The spatial distribution of air pollution estimates at the PCD level is illustrated in Appendix C Figure A1. (Appendix C Figure A2 reports the surfaces of annual estimates, as daily maps demonstrate wide variation depending on the day chosen for representation.) The air pollution surfaces were produced at the annual level (by averaging daily estimates) to demonstrate the spatial distribution of the estimated concentrations from daily LUR and IDW. Although having the same spatial resolution (i.e., PCD centroids), the surfaces generated from LUR models capture more spatial heterogeneity compared to the surfaces from IDW models. In rural areas, where there are fewer monitoring stations, the spatial variation is almost uniform when using IDW models.

**Figure 2:**
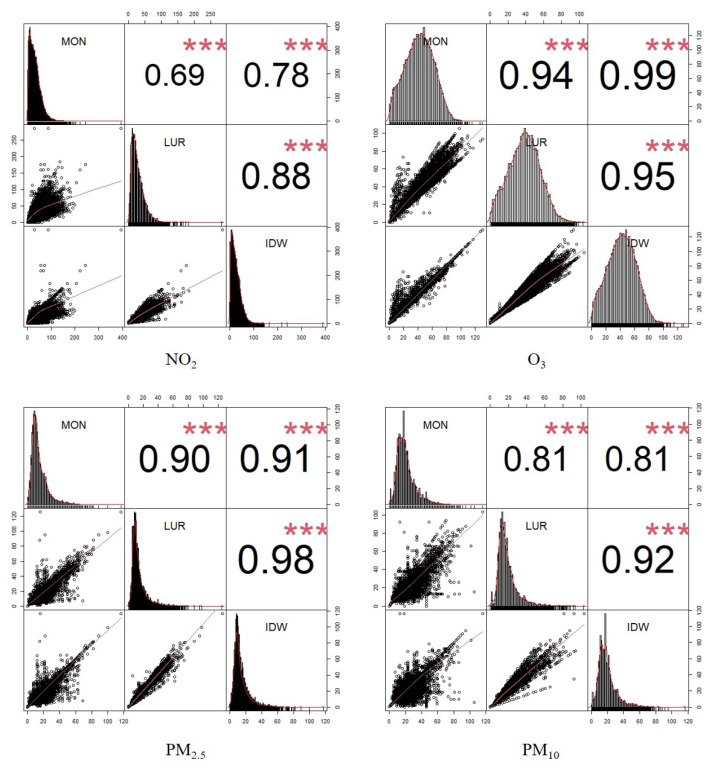
Pairwise scatter plots (bottom-left cells), histograms of concentrations’ distribution (left diagonal), and Person coefficients (top-right cells) for daily concentrations of ${\mathrm{NO}}_{2}$, $O_{3}$ (top row), ${\mathrm{PM}}_{2.5}$, and ${\mathrm{PM}}_{10}$ (bottom row). It shows paired comparisons of monitored daily concentrations (MONs), estimated daily concentrations based on LUR scaling (LUR), and estimated daily concentrations based on IDW (IDW). All correlations are significant. Note that this is a naive comparison at monitoring stations, which does not reflect the accuracy of estimates in out-of-sample areas as the IDW concentrations are entirely based on measurements from nearest monitors within a 50 km radius. Stars represent *p*-values: *** p<0.001.

Figure 2 compares daily air pollution concentrations using estimates from the daily LUR and IDW models with measurements. Overall, both modelling approaches were in high agreement with the measurements from monitoring stations—with the highest correlations observed for ${\mathrm{PM}}_{10}$, ${\mathrm{PM}}_{2.5}$, and $O_{3}$. IDW appeared more precise than, or equally as good as, daily LUR in this comparison. This was expected, as IDW models are completely informed by measurements at monitoring stations, and the comparison does not reflect the accuracy of estimates in out-of-sample areas (i.e., any locations other than monitoring stations). Furthermore, the IDW estimates of several locations used only one nearest monitoring station, which resulted in perfect fitting between measurements and estimates.

**Figure A3:**
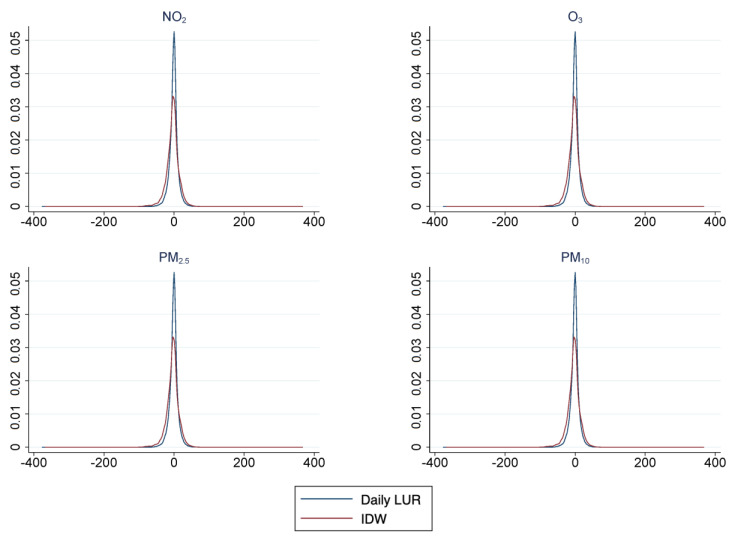
The density plots represent the differences between the daily LUR or IDW estimates and the air pollution concentration measured at monitoring stations, where the monitoring station itself has been removed from the calculation of the estimate.

Table 1 shows summary statistics that quantify the daily average difference across all four pollutants between the average daily LUR or IDW estimates and the average daily air pollution concentration observed at the different air pollution monitoring stations that were omitted in the calculation of the estimates. The magnitude of the deviation varies by pollutant, but on average, daily LUR provides more accurate estimates than IDW: the averages of the difference are smaller using daily LUR, for all pollutants, than IDW. The standard deviations of the difference are also smaller for daily LUR, suggesting greater accuracy of the estimates. In addition, IDW tends to provide maximum values that are larger than the maximum values obtained using daily LUR, which is true for all pollutants, except for ${\mathrm{NO}}_{2}$. This is further supported by looking at the distributions of the differences reported in Appendix D Figure A3 for each pollutant. Daily LUR appears to be more accurate than IDW.

Table 2 shows the model performance from the validation, where daily estimates were regressed against daily measurements at monitoring sites. Daily LUR outperformed IDW for all pollutants across all parameters. Briefly, daily LUR accounted for 13% to 39% more variation in the measured daily concentrations compared to IDW. Daily LUR also had lower RMSE values, which suggests smaller errors (RMSE ranging from 6.87 to 14.50 $\mu gm^{-3}$ for daily LUR; 8.21 to 22.80 $\mu gm^{-3}$ for IDW).

**Figure 3:**
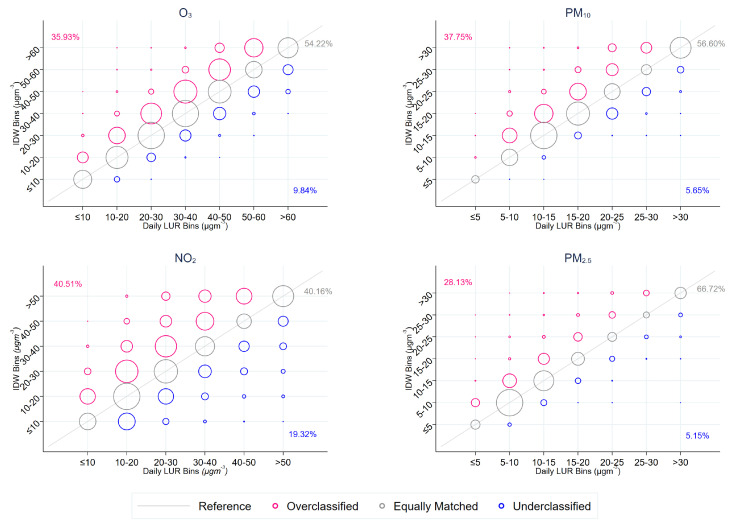
Scatter graphs comparing the number of visits classified to air pollution bins, defined using IDW and daily LUR techniques. Pollution concentrations are across four pollutants: ${\mathrm{NO}}_{2}$, $O_{3}$, ${\mathrm{PM}}_{10}$, and ${\mathrm{PM}}_{2.5}$. The size of the circle represents the number of visits. The percentage (%) match of air pollution indicator bins derived by IDW compared to air pollution indicator bins derived daily LUR estimates. A misclassified overestimated group is when IDW-estimated exposure is classified to be larger than those classified by daily LUR-estimated exposure. A misclassified underestimated group is when IDW exposure is classified to be smaller than those classified by the daily LUR-estimated exposure.

When assessing exposure by indicator bins, we found large discrepancies in the classification. Figure 3 illustrates the differences between the daily LUR and IDW bins for each pollutant. In our dataset, only 66.7% of observations fall into the same bin for ${\mathrm{PM}}_{2.5}$ across both exposure assignment techniques. This match decreases to 56.5% for ${\mathrm{PM}}_{10}$, 54.2% for $O_{3}$, and 40.2% for ${\mathrm{NO}}_{2}$. This suggests that, depending on the pollutant, at least 33.3% of the observations were inconsistently assigned to a pollutant bin. In some extreme cases, hospitals were assigned to high air pollution exposure bins in one method, but to low air pollution exposure bins in the other. While such exposure misclassification occurs, it appears that IDW is more likely to classify hospitals in higher exposure bins, relative to the daily LUR groups. This was expected as IDW relies on air pollution monitoring stations that are often located in areas of concern, where pollution concentrations are more likely to be high. Therefore, IDW extrapolates over extreme values over distances and, therefore, potentially overestimates air pollution exposure at hospitals.

### 5.2. Regression Results

In the previous section, we demonstrated that the classification of air pollution exposure using traditional indicator bins corresponded to, at best, 66% of observations being allocated to the same indicator bin when using daily LUR and IDW. Specifically, we observed that IDW measurements appeared to generally classify observations to higher exposure bins. In this section, we assess how this difference in classification impacts the estimated effects of air pollution on A&E visits using Equation (6).

In all cases, we found different point estimates when using daily LUR and IDW across comparable exposure bins. We found that the estimated changes, associated with air pollution exposure, to A&E utilisation rates varied depending on the air pollution exposure assessment approach applied. This variation differed across pollutants.

**Figure 4:**
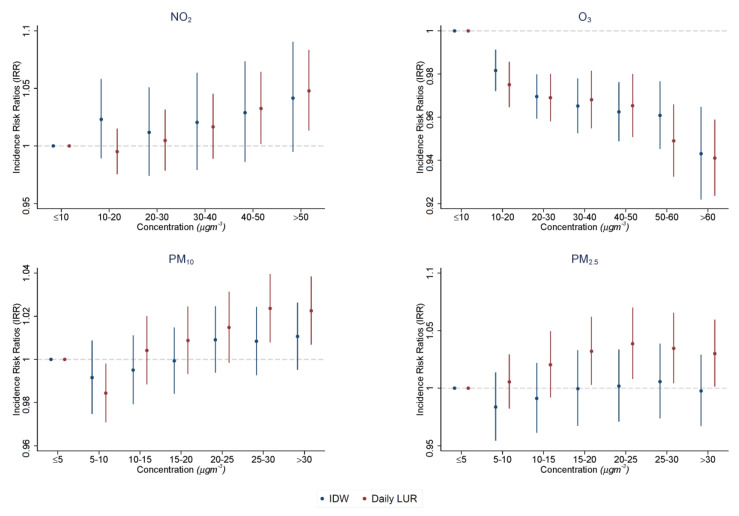
Incidence risk ratios (IRRs) of the effect of exposure on a day in a given range of each pollutant, relative to a day in the reference group. This represents the contemporaneous effect of each pollutant ($\beta_{p}$). For ${\mathrm{PM}}_{2.5}$ and ${\mathrm{PM}}_{10}$, estimates are shown across 5 $\mu gm^{-3}$ bands with a reference group in the 0–5 $\mu gm^{-3}$ range. For ${\mathrm{NO}}_{2}$ and $O_{3}$, estimates are shown across 10 $\mu gm^{-3}$ bands with a reference group in the 0–10 $\mu gm^{-3}$ range. Each dot represents the exponent of the point coefficients with 95% CIs reported by the lines on each side of estimates. The results presented are for air pollution exposure assignment through (a) IDW and (b) daily LUR.

When comparing the point estimates between exposure assessment approaches for ${\mathrm{NO}}_{2}$ (Figure 4), we obtained estimates of similar sizes. However, these estimates varied in statistical significance between exposure assignment techniques. We observed no statistically significant effect of air pollution on hospital visits across the pollutant when using IDW as the exposure assessment technique. This implies that there is no relationship between ${\mathrm{NO}}_{2}$ and A&E visits. Conversely, we saw a steady increase in hospital visits associated with ${\mathrm{NO}}_{2}$ exposure when using daily LUR as the exposure assessment approach. Statistical significance was observed for the more extreme air pollution bins, 40–50 $\mu gm^{-3}$ (*p* < 0.01) and >50 $\mu gm^{-3}$ (*p* < 0.001). The estimates for both methods are illustrated in Appendix E Table A3.

When comparing the point estimates between exposure assessment approaches for $O_{3}$ (Figure 4), we obtained estimates of similar sizes. Both exposure assessment techniques had estimates of statistical significance. In both instances, we observed a decrease in hospital visits as air pollution exposure increases. The estimates for both methods are illustrated in Appendix E Table A4.

${\mathrm{PM}}_{10}$ and ${\mathrm{PM}}_{2.5}$ display different relationships across exposure assessment approaches (Figure 4). For ${\mathrm{PM}}_{10}$, we saw a steady increase in visits as air pollution increases. However, this effect was statistically non-significant when using IDW. Conversely, there appeared to be statistically significant effects for values above 25 $\mu gm^{-3}$ using daily LUR. For ${\mathrm{PM}}_{2.5}$, point estimates varied in size between daily LUR and IDW, with only statistically significant results when using daily LUR for values above 15 $\mu gm^{-3}$. The regression results are outlined in Appendix E Table A5.

These estimates are plain correlations and by no means causal in these models. The nature of A&E visits are non-specific in our data, and therefore, the effects observed are likely masked by the aggregation to all-cause visits. The value of the different regressions only lies in the comparison of the coefficients using the different pollution assignment methods.

## 6. Conclusions

Ambient air pollution is an environmental factor with wide-ranging effects on human health and well-being. The assessment of air pollution exposure on social outcomes requires the estimation of air pollution, which has been performed in the economic literature in several ways. We illustrated how a widely used method in the social sciences, IDW, misclassifies air pollution concentrations, particularly in areas with sparse monitoring networks. We proposed a simpler computational approach, based on land use regression (LUR), that increases the geographical precision and accuracy compared to IDW, while still offering estimates of high temporal frequency. Our LUR outperformed IDW in our cross-validation study using various indicators of performance.

The difference in parameter estimates for the IDW approach and the daily LUR model was likely due to the inability of the IDW approach to account for different emission sources (such as road traffic, industrial activities) and topographies. We observed that, on average, air pollution concentrations derived from daily LUR showed smaller prediction errors than IDW and, thus, a higher accuracy. The instability of the IDW approach was also documented by [32], who compared this with a dispersion model to find the latter outperforming the inverse distance approach, when using annual air pollution concentrations. Whilst the use of dispersion models provides reliable air pollution estimates, their use is computationally demanding and generally inaccessible for wider contexts.

Our findings showed that the IDW approach, which has been the convention to measure air pollution in previous economic studies, is likely to exacerbate measurement error in exposure assignment due to its lower accuracy and precision. The level of these varies by pollutant. For PM, which comprises atmospheric aerosol particles that fluctuate less geographically compared to ${\mathrm{NO}}_{2}$ and travel long distances, both ${\mathrm{PM}}_{10}$ and ${\mathrm{PM}}_{2.5}$ displayed small discrepancies in their assigned air pollution exposure and, therefore, negligible differences in the estimated health impacts. For both pollutants, we failed to identify health impacts using IDW, otherwise observed with daily LUR. Contrastingly, ${\mathrm{NO}}_{2}$ is a pollutant that diffuses rapidly and, therefore, exhibits a higher degree of spatial variation. In this case, the two concentrations assigned using the two different methods were largely different, being in agreement for less than half of our observations. Although this resulted in similar point estimates of the impact of air pollution concentrations on the health outcome, the variability observed was much smaller under the daily LUR approach, which resulted in statistically significant health impacts. Finally, health estimates associated with $O_{3}$ were relatively unresponsive to exposure assessment approaches. Overall, the daily LUR model approach was able to account for some of the spatio-temporal variation associated with each pollutant, resulting in (i) the assignment of a more accurate and precise air pollution concentration and (ii) a more precise estimate of associated health impacts.

It is important to acknowledge that the economic significance of any variation created by the choice of pollution exposure method will vary with the pollution–outcome dose–response function related to the outcome of interest. In our illustration, the use of IDW resulted in an overestimation of air pollution effects on hospital utilisation, compared to the daily LUR. However, as other outcomes (e.g., mortality, obesity, productivity, etc.) carry their own unique relationship with air pollution, the associated sensitivity to exposure assignment may be of different magnitudes. In instances where large changes in air pollution are required to identify an impact on the outcome (e.g., obesity), the consequence of this difference in pollution exposure assignment may be smaller than in studies where small changes in air pollution are meaningful (e.g., mortality).

This paper illustrated how LUR models can be adapted to construct a reliable and frequent measure of local air pollution exposure. The daily LUR has several important advantages over other exposure assignment techniques, including less stringent data requirements, low computational costs, and the consideration of environmental characteristics, topological variation, and atmospheric conditions. Still, some of the emission sources and process characteristics used in the daily LUR model could be subject to imprecise measurement. While this approach is not devoid of measurement error, we have begun to bridge the gaps in accurate air pollution modelling for economic assessment. Most importantly, the availability of accessible LUR models for various cities and countries allows for this technique to be used in less-studied contexts (e.g., low- and middle-income countries with poorer and sparse monitoring networks).

These findings emphasise the need to be mindful of the exposure assessment technique utilised in economic studies as, depending on the pollutant, conventional approaches may introduce degrees of measurement error and variability that have the potential to bias the analysis and underestimate the impacts of air pollution. Our results may contribute to a more accurate evaluation of air pollution impacts and, subsequently, inform future environmental policies.

## Figures and Tables

**Table 1:** Summary statistics describing the absolute difference, in $\mu gm^{-3}$, between the average daily pollution observed at the monitoring site and average daily pollution concentrations at the same site estimated through (a) daily LUR or (b) IDW ($|Deviation_{m,d}|$). Pollution concentrations are across four pollutants: $O_{3}$, ${\mathrm{NO}}_{2}$, ${\mathrm{PM}}_{2.5}$, and ${\mathrm{PM}}_{10}$.

	Daily LUR					IDW				
	Mean	SD	5th	95th	N	Mean	SD	5th	95th	N
${\mathrm{NO}}_{2}$	10.09	10.42	0.70	28.39	21,535	14.07	12.17	1.00	35.00	21,535
${\mathrm{PM}}_{2.5}$	10.33	9.02	0.70	28.15	22,995	16.96	15.24	1.10	47.00	22,995
$O_{3}$	5.70	6.23	0.35	18.19	9,125	10.02	8.86	1.00	27.50	9,125
${\mathrm{PM}}_{10}$	4.25	5.39	0.28	12.68	13,870	5.51	6.08	0.30	16.40	13,870

**Table 2:** Model performance for daily LUR and IDW by regressing daily predictions against daily measurements. N: number of daily mean predictions; $R^{2}$: coefficient of determination; RMSE: root-mean-squared error (unit: $\mu gm^{-3}$).

	Daily LUR					IDW				
	N	*R* ^2^	RMSE	Beta	Intercept	N	*R* ^2^	RMSE	Beta	Intercept
${\mathrm{NO}}_{2}$	21,535	0.52	14.50	0.73	5.69	21,535	0.25	18.61	0.54	11.10
${\mathrm{PM}}_{2.5}$	13,870	0.56	6.87	0.81	2.78	13,870	0.43	8.21	0.74	5.23
$O_{3}$	9,125	0.59	13.72	0.82	11.52	9,125	0.20	22.80	0.42	28.61
${\mathrm{PM}}_{10}$	22,995	0.50	8.45	0.75	4.80	22,995	0.15	13.37	0.41	13.49

## Data Availability

Data can be obtained through application to the NHS. The authors do not have permission to share health data.

## Appendix A. Estimation of the Land Use Regression Model

**Stage 1: Identification of the “Land use” predictor variables:** Six types of Geographic Information System (GIS)-derived land use data were obtained including: land cover, population/household, road network, traffic, topography, and building. Potential predictor variables were characterised with different spatial scales by creating circular buffer zones around monitoring sites. The predictors were chosen mostly based on the ones used in the European Study of Cohorts for Air Pollution Effects (ESCAPE) study [49,60,64], with one predictor on building volume [61]. Each variable should be assigned a predefined direction of effect. The direction of effect was based on whether the variable acts as a source (+) or sink (−) and was used to guide the selection of predictor variables for modelling. For ${\mathrm{NO}}_{2}$, ${\mathrm{PM}}_{2.5}$, and ${\mathrm{PM}}_{10}$, variables for traffic, population, and the built environment (building, road, industry, etc.) were expected to increase air pollution concentrations (i.e., have a positive direction of effect). Vegetation land covers and open water, on the other hand, were expected to decrease pollution concentrations (i.e., negative direction of effect). The direction of effects was the opposite for $O_{3}$ because $O_{3}$ can be degraded by ${\mathrm{NO}}_{x}$.

Chemical transport model (CTM) estimates were also included as an additional predictor variable. CTM provides a three-dimensional complex simulation of air conditions. CTMs include formation, advection, deposition, and dispersion of air pollutants based on emission inventories (location, strength, size) and meteorological inputs (e.g., temperature, relative humidity, wind speed, and wind direction) [11]. CTMs have been increasingly used to predict the distribution of air pollution in large spatial domains. CTM estimates are usually available as a complete surface at various temporal and spatial resolutions. The CTM variable is obtained by extracting values at monitoring sites from the surface and, then, if the temporal resolution of the CTM surface is finer than annual, averaging to the annual mean.

**Stage 2: Annual LUR model**: A pilot study was first conducted to test the goodness of the fit of individual variables. Variables with zero values for a large number of monitoring sites (e.g., more than 80%) were removed. The modelling followed a standard approach for developing LUR models as described in [64]. The potential predictor variable with the highest correlation with all monitored concentrations was then offered to the model, followed by the next-ranking variable using a supervised forward stepwise method. A predictor variable was maintained in the final model if (i) the increment of the adjusted $R^{2}$ was greater than 1%, (ii) the coefficient conformed to the pre-determined direction of effect, and (iii) the p-value was no greater than 0.05. If the resulting model included the same variable with different buffers, the variable of less significance was removed. This was to avoid variables overlapping and make the models more intuitively interpretable [49].

The best combination of variables has the highest $R^{2}$. The output model contains a “best” set of predictor variables and associated coefficients (Equation (A1)).

(A1) $$y=b_{0}+b_{1}x_{1}+b_{2}x_{2}+\cdots +b_{p}x_{p}+\varepsilon$$

where y is the monitored annual average concentration, $b_{0}$ is the y-intercept (constant term), $b_{1};b_{2};\ldots ;b_{p}$ are the estimated regression coefficients, $x_{1};x_{2};\ldots ;x_{p}$ are the independent land use variables, and ε is the error term.

Models were checked for collinearity using the variance inflation factor (VIF) and spatial autocorrelation using Moran’s I of residuals. The VIF measures how much of the variance of the estimated coefficients is increased due to collinearity. If VIF > 3, the variable is to be removed from the model. Moran’s I coefficient is between −1 and +1, with 0 indicating no correlation with the nearby monitoring sites. Statistical analysis was performed in SPSS 24.0 [65].

Models were validated using *k*-fold cross-validation. Typically, one would use 5 or 10 folds depending on the data size. Our models included 26–63 monitoring sites; therefore, we used five folds in the analysis. The monitoring sites were randomly divided into five folds. One of the folds was used as the testing set, and the remainder acted as the training set. The operation iterates five times until all subsets are leaved out once. The model performance was then assessed on the predictions versus the monitored concentrations. This generated a squared Pearson correlation, which indicated the predictive ability of the models. Models were summarised by several measurements including adjusted $R^{2}$, root-mean-squared error (RMSE), and coefficient (beta). The formulae for the $R^{2}$ and RMSE are as follows:

(A2) $$R^{2}=1-\frac{sum\;squared\;regression(SSR)}{total\;sum\;of\;squares(SST)}=1-\frac{\sum {\left(y_{i}-{\hat{y}}_{i}\right)}^{2}}{\sum {\left(y_{i}-\bar{y}\right)}^{2}}$$

where ${\hat{y}}_{1},{\hat{y}}_{2},\ldots ,{\hat{y}}_{n}$ are predicted concentrations, $y_{1},y_{2},\ldots ,y_{n}$ are monitored concentrations, *n* is the number of monitoring sites, and $\bar{y}$ is the mean of monitored concentrations.

(A3) $$RMSE=\sqrt{\sum_{i=1}^{n}\frac{{\left({\hat{y}}_{i}-y_{i}\right)}^{2}}{n}}$$

where ${\hat{y}}_{1},{\hat{y}}_{2},\ldots ,{\hat{y}}_{n}$ are predicted concentrations, $y_{1},y_{2},\ldots ,y_{n}$ are monitored concentrations, and *n* is the number of monitoring sites.

The model performance of the annual LUR is presented in Appendix B Table A1.

**Stage 3: Annual air pollution surface**: An annual air pollution surface was created for each pollutant. The surface was built in ArcGIS v10.4 [66]. The resolution of the surface was determined by the smallest resolution of the datasets used.

Creating an air pollution surface involved four main steps: (i) creating a fishnet of cells covering the whole study area, (ii) extracting the values of the selected variables around the centroid of each cell, (iii) creating raster layers for the selected variables, and (iv) applying the constant and coefficients from the LUR model to the raster layers and compiling the surface.

## Appendix B. Descriptive Statistics

**Table A1:** Summary statistics for pollution concentrations obtained using LUR models.

	N *	Model adj. R^2^	CV-R^2^	RMSE	Variable	Buffer	Beta
NO_2_	86	0.762	0.736	0.343	(Constant)	-	1.808
					Road length	500	0.080
					MACC	-	0.320
					Traffic load	50	0.016
					Natural land	400	−0.019
PM_2.5_	53	0.770	0.631	0.137	(Constant)	-	1.507
					MACC	-	0.444
					Building volume	100	4.64 × 10^−4^
					Traffic load	50	3.27 × 10^−4^
					Natural land	1000	−0.003
PM_10_	41	0.696	0.540	0.175	(Constant)	-	0.591
					MACC	-	0.852
					Building volume	50	0.002
					Natural land	400	−0.018
					Traffic load	50	2.06 × 10^−4^
O_3_	65	0.816	0.799	4.944	(Constant)	-	7.622
					MACC	-	0.841
					Road length	500	−0.930
					Natural land	400	0.146

* Number of monitoring sites.

**Table A2:** Summary statistics of each air pollutant (in $\mu g/m^{3}$) across the various pollution bins used.

	IDW							Daily LUR						
	Mean	SD	Min	25th Quartile	75th Quartile	Max	N	Mean	SD	Min	25th Quartile	75th Quartile	Max	N
**Pollutant, $O_{3}$**														
<10	6.44	2.44	0	4.67	8.47	10	3914	5.87	2.61	0	3.80	8.11	10	5118
10–20	15.47	2.89	10	13	18	20	7751	15.26	2.96	10	12.61	17.79	20	9187
20–30	25.38	2.86	20	23	27.95	30	12,690	25.14	2.88	20	22.60	27.66	30	14,176
30–40	35.18	2.89	30	32.75	37.73	40	16,066	35.04	2.86	30.01	32.60	37.50	40	17,359
40–50	45.04	2.90	40	42.54	47.55	50	16,008	44.79	2.89	40.01	42.25	47.26	50	16,360
50–60	54.83	2.89	50	52.26	57.17	60	11,924	54.49	2.84	50	52.03	56.82	60	10,345
>60	69.16	7.57	60	63.46	73	131	11,947	68.23	7.19	60	62.77	71.97	110.54	7755
Overall	40.27	18.20	0	27.02	53	131	80,300	36.87	17.32	0	24.29	48.68	110.54	80,300
**Pollutant, ${\mathrm{NO}}_{2}$**														
<10	6.11	2.73	0	4	8.62	10	10,708	7.26	2.10	0	6.02	8.95	9.99	9795
10–20	15.22	2.84	10	12.95	17.73	20	18,409	14.93	2.89	10.01	12.48	17.39	19.99	25,255
20–30	24.95	2.85	20	22.55	27.33	30	18,146	24.68	2.87	20	22.15	27.13	29.99	19,699
30–40	34.82	2.86	30	32.29	37.19	40	13,919	34.48	2.86	30	31.98	36.85	40	12,435
40–50	44.59	2.88	40	42.01	47	50	9201	44.44	2.83	40.01	42.01	46.82	49.99	6741
>50	62.49	11.64	50	54.09	67.50	145	9917	64.64	16.44	50	53.80	69.19	245.35	6375
Overall	28.80	17.69	0	15.25	39.20	145	80,300	25.84	16.39	0	14	33.66	245.35	80,300
**Pollutant, ${\mathrm{PM}}_{2.5}$**														
<5	4.09	0.96	0	3.71	5	5	3796	4.04	0.83	0	3.69	4.68	5	6642
5–10	8	1.36	5	7	9	10	28,446	7.55	1.36	5.01	6.43	8.66	10	32,086
10–15	12.28	1.43	10	11	13.44	15	21,307	12.16	1.42	10.01	10.93	13.29	14.99	19,643
15–20	17.36	1.43	15	16	18.59	20	11,013	17.24	1.43	15	15.98	18.48	20	9207
20–25	22.37	1.45	20	21	23.64	25	6171	22.24	1.45	20.01	20.94	23.51	24.99	5098
25–30	27.42	1.44	25	26.10	28.58	30	3499	27.26	1.39	25	26.07	28.44	29.99	2710
>30	40.71	9.14	30	33.55	45.49	100	6068	40.03	8.72	30.01	33.66	44.20	106.28	4914
Overall	14.66	9.72	0	8.41	17.89	100	80,300	13.08	9.17	0	7.25	15.79	106.28	80,300
**Pollutant, ${\mathrm{PM}}_{10}$**														
<5	2.13	0.60	2	2	2	5	1358	1.88	0.83	1.51	1.60	1.71	4.99	1422
5–10	8.81	1.19	5.01	8	10	10	6074	8.40	1.18	5.01	7.66	9.38	10	12,017
10–15	12.87	1.35	10	12	14	15	20,493	12.41	1.40	10	11.24	13.58	15	23,469
15–20	17.66	1.40	15	16.39	19	20	20,210	17.31	1.41	15	16.02	18.50	20	17,816
20–25	22.55	1.45	20	21.04	24	25	12,797	22.26	1.43	20	21.05	23.43	25	11,004
25–30	27.58	1.48	25	26.01	29	30	6658	27.30	1.45	25	26.02	28.55	30	5458
>30	41.51	10.74	30	33	47	116	12,710	40.60	10.05	30	32.98	44.89	114.19	9114
Overall	20.88	11.28	2	13.22	25	116	80,300	18.27	10.35	1.51	11.42	22.09	114.19	80,300

## Appendix E. Regression Tables

**Table A3:** Incidence risk ratios (IRRs) of the effect of exposure on a day in a given range of ${\mathrm{NO}}_{2}$, relative to a day in the 0–10 $\mu gm^{-3}$ range. This represents the contemporaneous effect of ${\mathrm{NO}}_{2}$ ($\beta_{p}$). A value of 1 represents no change. Estimates are shown for two pollution exposure assignment methods: inverse distance weighting (IDW) and daily land use regression (daily LUR).

	${\mathrm{NO}}_{2}$	
	IDW	Daily LUR
10–20	1.023	0.995
	(0.018)	(0.010)
20–30	1.012	1.005
	(0.020)	(0.014)
30–40	1.020	1.017
	(0.022)	(0.014)
40–50	1.029	1.033 *
	(0.022)	(0.016)
>50	1.042	1.048 **
	(0.024)	(0.018)
Day of Week FE	Yes	Yes
Bank Holidays FE	Yes	Yes
School Holidays FE	Yes	Yes
Month FE	Yes	Yes
Hospital FE	Yes	Yes
3-Day Lags	Yes	Yes
Mean Relative Humidity	Yes	Yes
Mean Temperature	Yes	Yes
Pseudo $R^{2}$	0.815	0.816
Observations	70,825	70,825

Exponentiated coefficients representing IRR. Robust S.E. clustered by NHS hospital in parentheses. * *p* < 0.05, ** *p* < 0.01.

**Table A4:** Incidence risk ratios (IRRs) of the effect of exposure on a day in a given range of $O_{3}$, relative to a day in the 0–10 $\mu gm^{-3}$ range. This represents the contemporaneous effect of $O_{3}$ ($\beta_{p}$). A value of 1 represents no change. Estimates are shown for two pollution exposure assignment methods: inverse distance weighting (IDW) and daily land use regression (daily LUR).

	$O_{3}$	
	IDW	Daily LUR
10–20	0.982 ***	0.975 ***
	(0.0049)	(0.0054)
20–30	0.970 ***	0.969 ***
	(0.0052)	(0.0056)
30–40	0.965 ***	0.968 ***
	(0.0065)	(0.0068)
40–50	0.962 ***	0.965 ***
	(0.0070)	(0.0075)
50–60	0.961***	0.949 ***
	(0.0080)	(0.0085)
>60	0.943 ***	0.941 ***
	(0.011)	(0.0090)
Day of Week FE	Yes	Yes
Bank Holidays FE	Yes	Yes
School Holidays FE	Yes	Yes
Month FE	Yes	Yes
Hospital FE	Yes	Yes
3-Day Lags	Yes	Yes
Mean Relative Humidity	Yes	Yes
Mean Temperature	Yes	Yes
Pseudo $R^{2}$	0.815	0.815
Observations	70,825	70,825

Exponentiated coefficients representing IRR. Robust S.E. clustered by NHS hospital in parentheses. *** *p* < 0.001.

**Table A5:** Incidence risk ratios (IRR) of the effect of exposure on a day in a given range of PM, relative to a day in the 0–5 $\mu gm^{-3}$ range. This represents the contemporaneous effect of PM ($\beta_{p}$). A value of 1 represents no change. Estimates are shown for ${\mathrm{PM}}_{2.5}$ & ${\mathrm{PM}}_{10}$. Estimates are shown for two pollution exposure assignment methods: inverse distance weighting (IDW) and daily land use regression (daily LUR).

	${\mathrm{PM}}_{2.5}$		${\mathrm{PM}}_{10}$	
	IDW	Daily LUR	IDW	Daily LUR
5–10	0.984	1.005	0.992	0.984 *
	(0.015)	(0.012)	(0.0087)	(0.0069)
10–15	0.991	1.020	0.995	1.004
	(0.015)	(0.015)	(0.0081)	(0.0081)
15–20	1.000	1.032 *	0.999	1.009
	(0.017)	(0.015)	(0.0078)	(0.0080)
20–25	1.002	1.039 *	1.009	1.015
	(0.016)	(0.016)	(0.0078)	(0.0084)
25–30	1.006	1.035 *	1.008	1.024 **
	(0.017)	(0.016)	(0.0081)	(0.0081)
>30	0.998	1.030 *	1.011	1.023 **
	(0.016)	(0.015)	(0.0079)	(0.0080)
Day of Week FE	Yes	Yes	Yes	Yes
Bank Holidays FE	Yes	Yes	Yes	Yes
School Holidays FE	Yes	Yes	Yes	Yes
Month FE	Yes	Yes	Yes	Yes
Hospital FE	Yes	Yes	Yes	Yes
3-Day Lags	Yes	Yes	Yes	Yes
Mean Relative Humidity	Yes	Yes	Yes	Yes
Mean Temperature	Yes	Yes	Yes	Yes
Pseudo $R^{2}$	0.815	0.815	0.815	0.815
Observations	70,825	70,825	70,825	70,825

Exponentiated coefficients representing IRR. Robust S.E. clustered by NHS hospital in parentheses. * *p* < 0.05, ** *p* < 0.01
